# Evaluation of the Efficacy of Magnetic Resonance Imaging for Feminizing Gender Affirmation Genital Surgery: A Novel Approach

**DOI:** 10.7759/cureus.60823

**Published:** 2024-05-22

**Authors:** Vinoth Vaithiya, Saravanakumar Subbaraj

**Affiliations:** 1 General Surgery, All India Institute of Medical Sciences, Madurai, Madurai, IND; 2 General Surgery, Mahatma Gandhi Medical College and Research Institute, Sri Balaji Vidyapeeth (Deemed to be University), Puducherry, IND

**Keywords:** gender dysphoria (gd), magnetic reasoning imaging (mri), vaginoplasty, gender affirmation surgery, transwomen, amab

## Abstract

Background

Gender dysphoria is treated with gender affirmation surgery (GAS) for assigned male at birth (AMAB) individuals. This study aimed to evaluate the postoperative anatomical changes in AMAB individuals who underwent GAS using magnetic resonance imaging (MRI) and to compare it with cis-females, thereby assessing the efficacy of the surgical technique in achieving pelvic anatomy similar to cis-females.

Methodology

This was a prospective observational study done in a tertiary care hospital. AMAB individuals who underwent gender affirmation genital surgery using single-stage solely penile skin inversion vaginoplasty were included after informed consent and approval by the Institutional Human Ethics Committee. Patients with complications such as deep space surgical site infection (SSI) and neo-vaginal prolapse were excluded. All the study participants were advised a vaginal self-dilatation regimen, reviewed three months after the surgery, and subjected to an MRI of the pelvis with a vaginal tutor. Parameters such as neo-vaginal depth, alpha (α) angle, rectovaginal thickness, and remnant of corpora cavernosa were measured and compared with cis-female parameters measured from images in the archives from the Department of Radiology.

Result

A total of 21 patients were included in the study, with a mean age of 27±4.7. Between the study group and cis-females, no significant difference was seen in vaginal depth, and cis-females had significantly higher values in other parameters. There was a significant difference between the subgroups, i.e., defaulters and non-defaulters in soft tissue parameters such as vaginal depth (p=0.001), α angle (p=0.002), and rectovaginal thickness (p=0.002) with the non-defaulter patients having higher values.

Conclusion

Single-stage penile skin inversion vaginoplasty is capable of producing anatomical parameters, importantly neo-vaginal depth, which is fairly comparable with cis-female, as evident in the non-defaulter subgroup patients. Proper compliance with the vaginal dilatation regimen plays a significant role in the maintenance of soft tissue pelvic anatomical parameters.

## Introduction

Gender dysphoria (GD) is the state of distress that is caused by incongruence between the gender assigned at birth and the self-identified gender of the individual. Those who are assigned male at birth (AMAB) and transformed to a more feminine role are called transwomen [1]. Gender affirmation surgery (GAS) describes a group of surgical procedures performed to change primary and/or secondary sexual characteristics to affirm a person's gender identity. Genital surgery is considered the final phase of the treatment for GD and an effective modality of treatment for appropriately selected individuals [2]. As per the latest census survey conducted by the government of India in 2011, the total population of transgender and gender-diverse (TGD) persons was around 4.9 lakhs [3]. However, this is very much an underestimated value.

Genital surgery has various components, among which vaginoplasty is an important part. Among various techniques for vaginoplasty, penile skin inversion vaginoplasty is considered the most preferred method [4]. Vaginal contracture is the most frequently observed late complication [5]. Magnetic resonance imaging (MRI) is one of the imaging modalities used for the assessment of neo-anatomy of post-operative patients. Complications such as neo-vaginal contracture once established will require further surgery to reverse. Hence, it needs early identification. MRI can be used for the assessment of compliance to post-operative advice and evaluation of postoperative changes effectively. There are only a limited number of studies present that have used MRI as a tool for the assessment of GAS patients.

In this study, we focus on the effectiveness of MRI in the evaluation of postoperative changes in transwomen (AMAB individuals) and the effectiveness of the surgical technique in achieving anatomical parameters that are comparable to cis-women.

## Materials and methods

**Figure 1:**
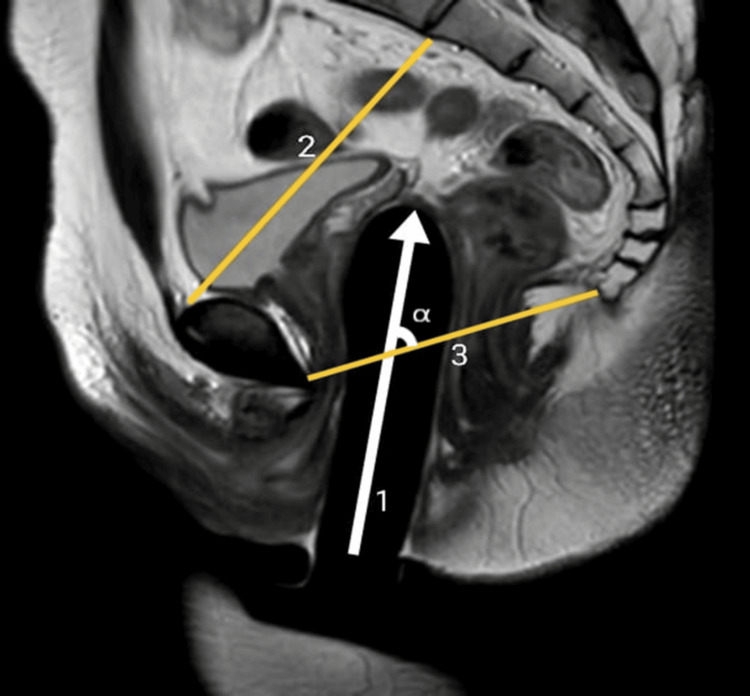
T2-weighted TSE sagittal MRI image of the post-operative patient Original MRI image of a patient who underwent the study. TSE - Turbo spin-echo; MRI - Magnetic resonance imaging; 1 - Neovaginal depth; 2 - Inlet of the pelvis (IOP); 3 - Inferior pelvic aperture (IPA); α - Alpha angle

**Figure 2:**
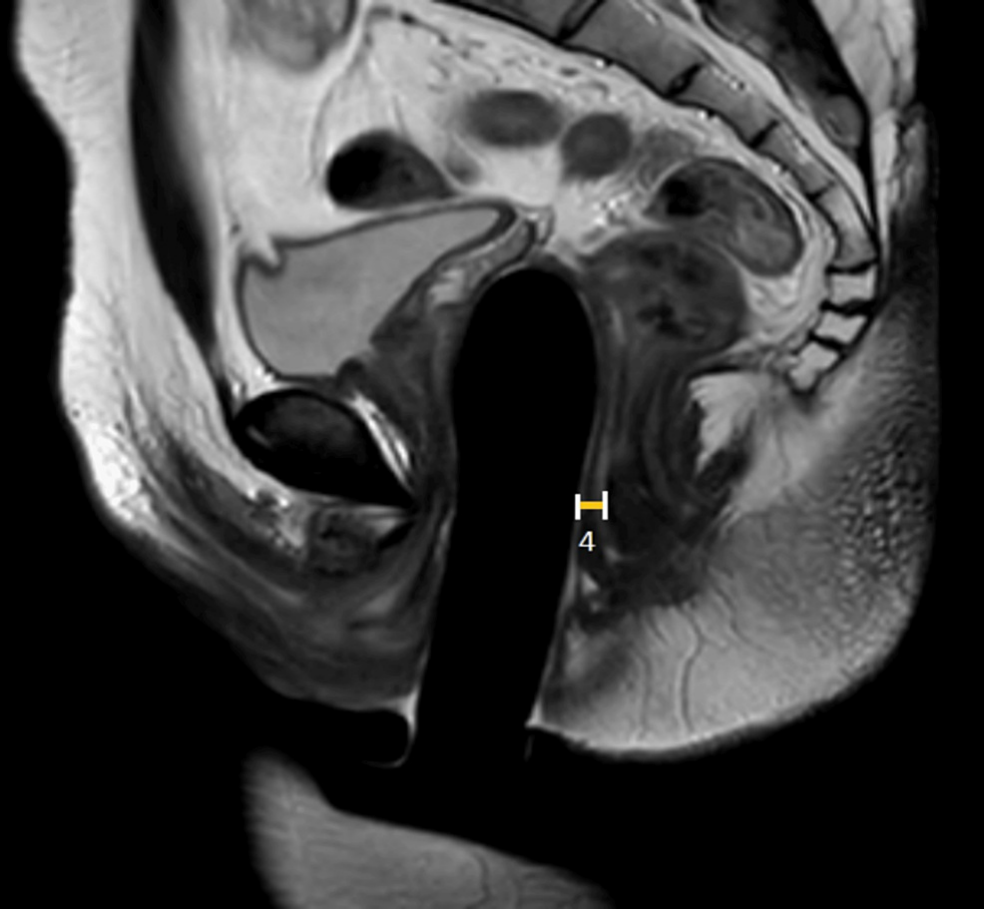
T2-weighted TSE sagittal MRI image of the post-operative patient Original MRI image of a patient who underwent the study. 4 - Rectovaginal thickness

This study was a prospective observational study done in a tertiary care centre in Pondicherry, India, between March 2021 to January 2023 after obtaining ethical approval from Institutional Human Ethics Committee, Mahatma Gandhi Medical College and Research Institute (MGMCRI), Pondicherry, India (approval number: MGMCRI/Res/01/2020/30/IHEC/345). The study subjects were AMAB individuals who presented to the tertiary centre and underwent gender affirmation genital surgery. All patients underwent single-stage GAS using solely the penile skin inversion vaginoplasty technique performed by the same well-experienced surgeon. Complete dissection of the penis was done till the root of the penis, and penectomy was performed. The neovaginal cavity is created in the pre-rectal space by blunt dissection of Denonvillier’s fascia. Postoperatively, the patients were prescribed a vaginal self-dilatation regimen as per standard of care guidelines [6]. AMAB individuals of age more than 18 years who underwent GAS were included after three months of the postoperative period, and patients with complications such as major surgical site infection (SSI) and neovaginal prolapse were excluded. Patients who followed the standard of care self-dilatation regimen were considered non-defaulters, and patients with poor/noncompliance were considered defaulters. After assessment and obtaining written consent, 21 patients were included in the study and were subjected to an MRI of the pelvis only once and three months after the surgery after the insertion of a silicon tutor in the neovagina. No oral or intravenous contrast agent was administered for the MRI assessment. Images were acquired from sagittal, coronal, and axial planes in T1-, T2-, and fluid-attenuated inversion recovery (FLAIR)-weighted sequences using the turbo spin-echo (TSE) technique. In all patients, the MRI examination was performed with a vaginal tutor. The vaginal tutor was inserted till the patient was comfortable and was able to tolerate it. Then, the following postoperative radiological anthropometric parameters were evaluated: (a) depth of the neovagina - the distance between the tip of the vaginal prosthesis and the skin surface of the neovaginal opening (Figure 1); (b) α angle - the angle between the neovaginal axis and inferior pelvic aperture (IPA) axis (Figure 1); (c) thickness of the rectovaginal septum measured at the middle part of the neovagina (Figure 2); and (d) remnants of crura of the corpora cavernosa.

**Figure 3:**
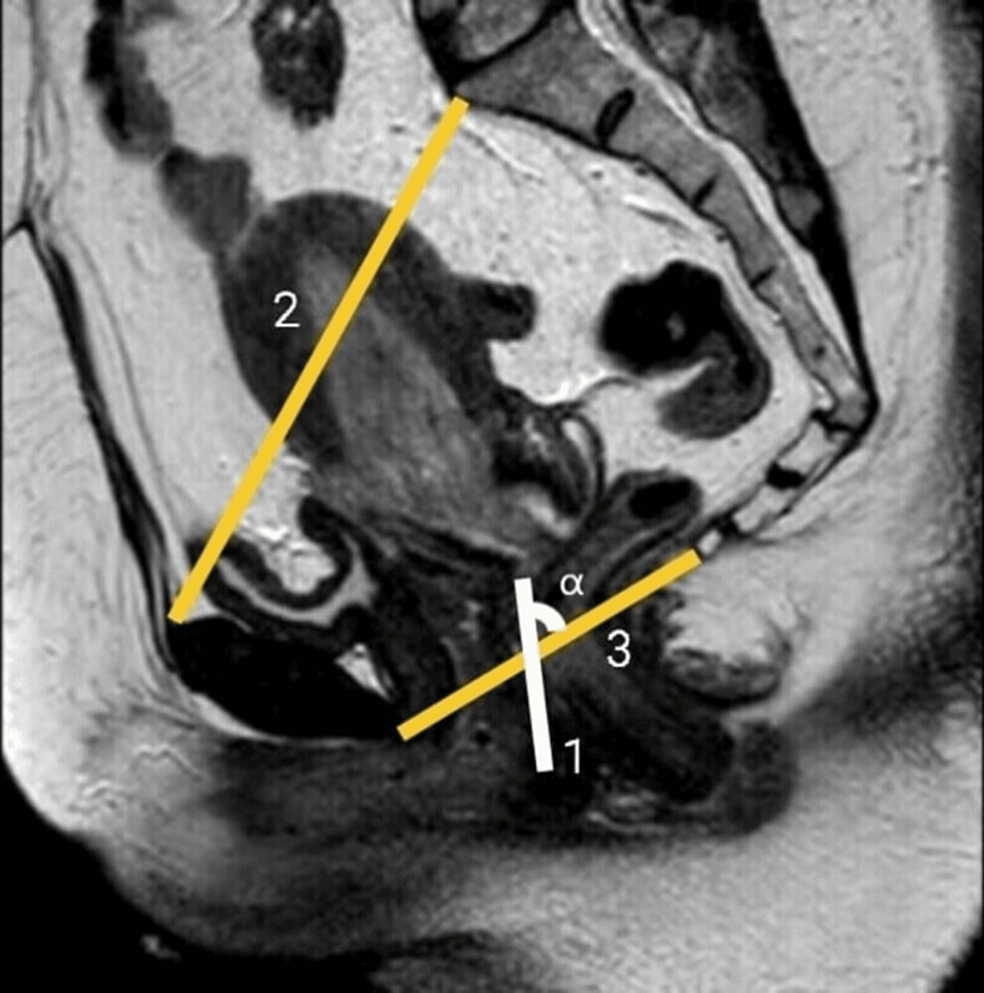
T2-weighted TSE sagittal MRI image of the normal cis-female Original MRI image of a cis-female patient taken from the archive. 1 - Neovaginal depth; 2 - Inlet of the pelvis (IOP); 3 - Inferior pelvic aperture (IPA); α - Alpha angle

The same parameters were measured from MRI images of age-matched (range: 20-40 years of age) ciswomen in the archives of the Radiology Department (Figure 3). The MRI images of the cis-women who had disease-altering pelvic anatomy were excluded.

## Results

Among the 21 patients analyzed, the majority 10 (48%) were between 20 and 25 years of age. Five (24%) of patients were between 26 and 30 years of age, and six (28%) were between 31 and 40 years of age, with an SD of 27±4.7. Among the 21 patients, 12 (57%) followed the vaginal self-dilatation regimen properly. The remaining nine (43%) were defaulters. Table 1 shows the values of all anthropometric parameters that were assessed in the study group, and cis-female values measured from the images in the archives are shown for comparison. Table 2 shows the comparison between defaulters and non-defaulters. There was a significant difference (p=0.001*) in vaginal depth between defaulters and non-defaulters. The compliant patients had a longer vaginal length than the defaulters. There was no significant difference between the study group and the cis-female (p=0.26) regarding vaginal depth. The vaginal axis (i.e., the alpha angle) was higher in non-defaulters. The defaulters had a lesser alpha angle, which was significant (p=0.002*). This shows a significant vaginal axis change in defaulters, and there was a significant difference between the study group and the cis-female group (p=0.0002*). This higher difference is due to the reduction of the alpha angle in the defaulter subgroup (37.49°±15). The rectovaginal thickness was also higher in non-defaulter patients, which reached significance (p=0.002*) when compared with defaulters. In comparison with cis-female parameters, there was a significant difference (p=0.0005*) between the study group and cis-females. This can also be due to the reduced thickness of the septum in the defaulter subgroup (0.61±0.19). In all 21 patients, no significant remnant of the corpora cavernosa was found.

**Table 1:** Measurement values for anthropometric parameters *p value less than 0.05 is significant.

Parameters	Study population			Cis-female (controls)			p value
	Range	Mean	Standard deviation	Range	Mean	Standard deviation	
Vaginal depth (cm)	8–11	9.07	±2.16	6-9	8.1	± 0.74	0.26
Alpha angle (degree)	10°–71°	50.1°	±18.27°	50°-70°	59.3°	± 4.2°	0.0002*
Recto-vaginal thickness (cm)	0.2–1.1	0.8	±0.22	1.2-2.8	1.92	± 0.45	0.0005*

**Table 2:** Subgroup anthropometric parameter analysis *p value less than 0.05 is significant.

Parameters	Subgroup	Range	Mean	Standard deviation	p value
Vaginal depth (cm)	Non-defaulters	9–11	9.94	± 0.63	0.001*
	Defaulters	8–9.3	8.41	± 1	
Alpha angle (degree)	Non-defaulters	51°–71°	60.86°	± 5.73°	0.002*
	Defaulters	10°–56°	37.49°	± 15°	
Recto-vaginal thickness (cm)	Non-defaulters	0.8–1.1	0.96	± 0.13	0.002*
	Defaulters	0.2–0.8	0.61	± 0.19	

## Discussion

The sexual and psychological well-being of patients after GAS surgery depends upon the aesthetic and functional outcome of the vaginoplasty. The main aim of GAS in AMAB individuals is to make the genital complex as feminine as possible in both appearance and functionality [7]. Over the period of time, the technique for GAS has changed drastically. Earlier, it was performed as a multistage procedure where penectomy/orchidectomy/vulvoplasty was done in the first stage and vaginoplasty was done in the second stage. Different techniques and their modifications were proposed and practiced in the late 19th century. Currently, pedicled penile skin inversion vaginoplasty is the preferred technique due to lesser post-operative complications. The usage of perineal/scrotal skin along with penile skin for neovaginal lining is not done in this study. Even though they can increase the depth, complications such as flap rejection, hair growth, etc. in the postoperative period make them less chosen options among the patients [8].

Age distribution

In this study, the mean age of the study population was 27±4.7 years, with a majority (15, 72%) being between 20 and 30 years and the remaining 6 (28%) being of the age group 31-40. The mean age of transwoman patients approaching medical care was 25.22 in Iran as per Talaei et al. [9]. This study also shows the majority of AMAB individuals seeking gender affirmation medical care were also in the 20-30 years age group. When compared to similar studies by Cova et al. in 2003 (mean age: 31) [10], Trombetta et al. [11] (mean age: 31) and Bertolotto et al. [12] (mean age: 36±10), this study had a younger population (mean age: 27±4.7), similar to the study population of Brunocilla et al. [13].

Neo-vaginal depth

**Figure 4:**
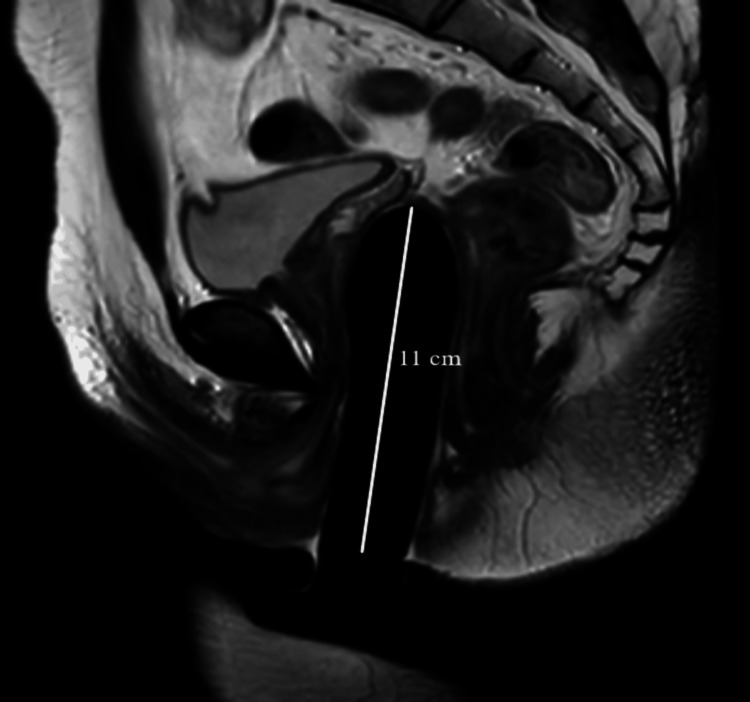
T2W HR sagittal image of a non-defaulter patient Original MRI image of a patient who underwent the study. T2W - T2 Weighted; HR - High resolution

**Figure 5:**
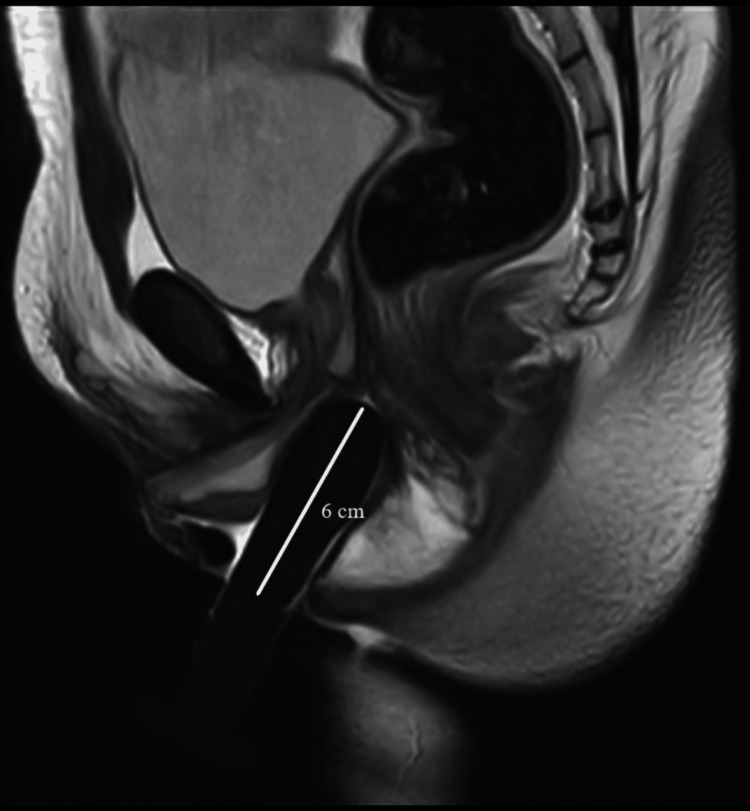
T2W HR sagittal image of defaulter patients Original MRI image of a patient who underwent the study.

In this study, the MRI was taken after the insertion of the silicone tutor because, without a tutor, the vaginal canal appeared smaller in diameter and shorter than the actual depth. The average vaginal depth of the study group (9.07±2.16 cm) was higher than cis-females (8.1±0.74). However, this difference was not significant (p=0.26), showing that adequate neovaginal depth was achieved in all patients of the study group. However, the mean value of the study population (9.07 cm) was found to be surprisingly higher than cis-females (8.1 cm). The cis-female images were taken from archives, and the MRI was done without a vaginal tutor. This could be a possible cause for reduced values of vaginal depth in cis-females. Twelve (57%) patients who adhered to the vaginal dilation as per the regimen were considered “non-defaulters,” and nine (43%) who failed/deviated from the vaginal dilation regimen are considered “defaulters.” Based on this, we had two subgroups in the study population. The average depth of neovagina in the non-defaulter group was 9.94±0.63 cm. However, in defaulter patients, the mean value of vaginal depth was 8.41±1 cm. Even though both patient groups were operated on using the same single-stage solely penile skin inversion technique and a minimum of 10 cm depth was achieved intraoperatively, there was a significant difference (p=0.001*) in vaginal depth between the two groups. MRI pelvis images of non-defaulter and defaulter patients are shown in Figure 4 and Figure 5, respectively.

This proves the importance of vaginal self-dilation in maintaining vaginal depth postoperatively. The postoperative MRI imaging of the pelvis gives us an opportunity to detect vaginal contracture early, for example, at three months in this study. The reasons for non-compliance with vaginal dilation in the defaulter subgroup were due to the lack of privacy and financial constraints among the patients.

The commonest late complication with penile skin inversion vaginoplasty is vaginal contracture and vaginal atresia [14]. Once vaginal contracture has occurred, it requires further surgery. The majority of these complications occur in the first four months of the postoperative period [15]. These complications are detected only at a later stage using the conventional clinical examination. From this study, we see that MRI has the ability to detect vaginal contracture early in defaulters when compared to conventional depth measurement using a dilator, which is subjective and can differ according to the expertise of the examiner.

Adequate vaginal depth is necessary for the postoperative satisfactory sexual life. A retrospective study by Massie et al. proved that the quality of life of these patients improved postoperatively when they gained sexual activity [16].

Based on the study, we conclude that proper compliance to the vaginal dilatation regimen results in neovaginal depth, which is fairly comparable to cis-female parameters, and solely penile skin inversion technique vaginoplasty is efficient in achieving adequate vaginal depth as proved in various studies [14].

Alpha angle

**Figure 6:**
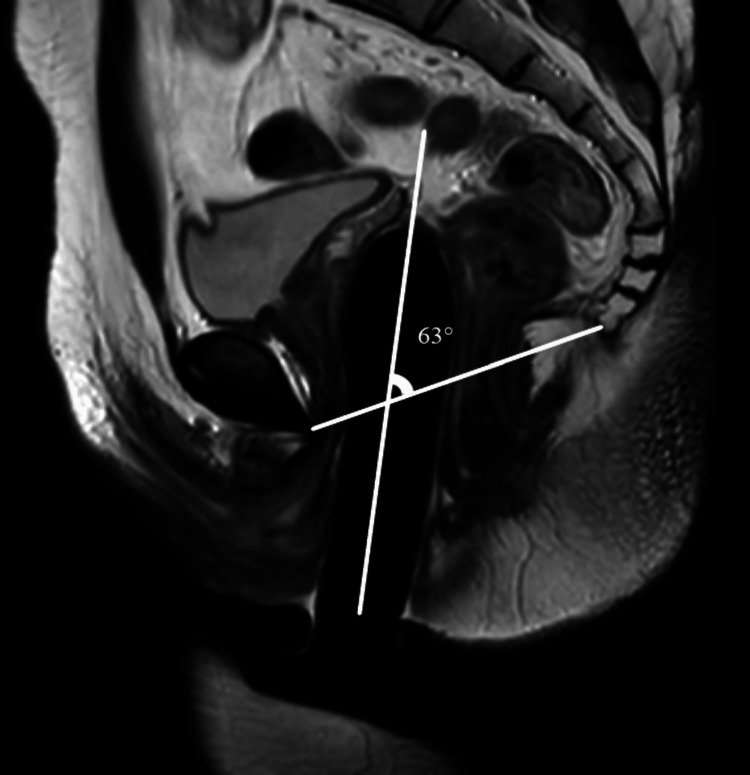
T2W HR sagittal image of a non-defaulter patient Original MRI image of a patient who underwent the study.

**Figure 7:**
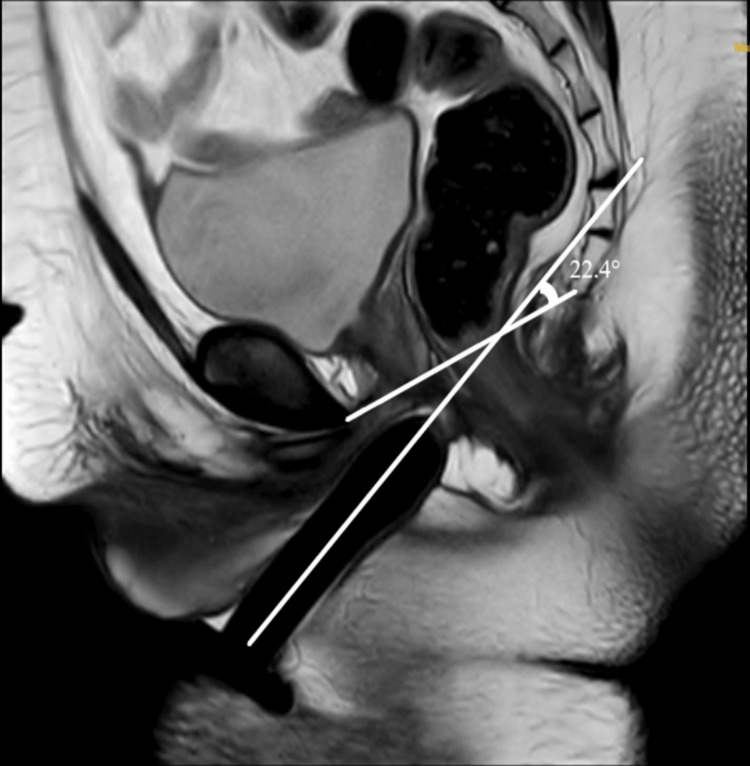
T2W HR sagittal image of a defaulter patient Original MRI image of a patient who underwent the study.

Apart from the depth, the axis of the neovagina plays a significant role in comfortable sexual intercourse [13]. Acute angulation of the neovaginal will cause dyspareunia for the patients postoperatively. The mean value of the alpha angle study population was 50.1°±18.27°, and the range was 10°-71°. There was a gross difference in the range and mean values between the two subgroups of the study population. The range of vaginal axis measured in non-defaulters was 55°-71° with a mean value of 60.86°± 5.73°. However, in defaulters, the range was 10°-50°, with a mean value of 37.49°±15° with a significant difference between the two subgroups (p=0.002*). MRI pelvis images of non-defaulter and defaulter subgroup patients were shown in Figure 6 and Figure 7, respectively.

Even though the vaginal depth of 8.6 cm was achieved in the defaulter patient, the vaginal axis was completely altered. Fibrosis of the pre-rectal space due to improper compliance with the dilatation regimen resulted in the reduction of alpha angle. When compared with the values obtained by Brunocilla et al. [13], the vaginal axis achieved in their study was 54°±11°, ranging within 30°-70°, similar to the non-defaulter subgroup of this study.

The cis-female mean value of the alpha angle was 59.3°±4.2° and ranged between 50° and 70°. When the study group was compared with cis-females, there was a significant difference (p=0.002*). This is due to the formation of a more acute angle in the defaulter subgroup (mean: 37.49°±15°). The mean value and range of non-defaulter patients were fairly comparable with normal cis-females with no significant difference (p=0.3). However, there was a significant difference between defaulters and cis-female values (p=0.001*). Based on the study, it is proven that self-dilatation of the neovagina is essential for the prevention of vaginal atresia thereby maintaining the vaginal axis at an optimum angle, which is fairly comparable and similar to the normal cis-female vaginal axis, as seen in the non-defaulter subgroup.

Rectovaginal thickness

Sufficient soft tissue thickness is necessary for the proper functioning of the vaginal canal. Various studies proved that adequate rectovaginal thickness is essential for the desirable functioning of the neovagina in the postoperative period [17]. In case of inadequate thickness, it will result in painful coitus and improper functionality of the vagina, causing reduced satisfaction for patients in their life post-surgery [18].

In this study, the mean value of the rectovaginal thickness for the study group was 0.8±0.22 cm with a range of 0.2-1.1 cm. The cis-female values measured from archives had a mean value of 1.92±0.45 cm (range: 1.2-2.8 cm) when compared to cis-females, there was a significant difference (p=0.0005*).

This is because the vagina of cis-females has three layers anatomically. They are the mucosal layer, the muscular layer containing two layers of circular smooth muscles, and the serosa layer [19]. All these layers are absent in the study group as the latter has only penile skin in the neovagina.

Remnant of corpora cavernosa

Various surgical modifications have been developed over the past century regarding the level of a penectomy. In the early period, the penectomy was done to the pubic bone or even distal to it, resulting in significant retention of corpora cavernosa. In a study conducted by Hage et al. [20], they found that patients with significant retention of corpora cavernosa either unknowingly or as a part of surgical technique performed by various surgeons had dyspareunia due to enlargement of erectile tissue during sexual arousal [20]. It is also observed in patients where complete penectomy was performed that they were well satisfied with their sexual life [8,21]. As the penectomy was done at the root of the penis, resulting in the complete removal of the erectile tissue, none of the patients from the study group had remnants of corpora cavernosa.

Limitations

The study group was relatively small. Only 21 patients were included in the study because of the COVID-19 pandemic during the study period. The cis-female images were taken from the archives of the Department of Radiology. Thus, imaging after the insertion of a vaginal tutor as performed in the study group was not possible.

## Conclusions

The postoperative soft tissue changes seen in AMAB individuals undergoing GAS were influenced greatly by the status of compliance to the vaginal self-dilation regimen, causing a significant difference between non-defaulter and defaulter patients. In non-defaulter patients, the postoperative parameters were well within the acceptable range and were fairly comparable to normal cis-female parameters. The surgical technique of sole penile skin inversion vaginoplasty is capable of producing pelvic soft tissue parameters, which are similar to cis-female values, provided that the vaginal dilation regimen is followed properly. MRI evaluation of these patients helped identify postoperative changes, complications at an earlier stage, and the status of the neo-vaginal axis, which can be missed in routine clinical examinations. This proves the superiority of imaging studies, especially MRI in the assessment and evaluation of patients in the postoperative period for early detection and further management of complications accordingly.
